# What Does Influence the Neonatal Microbiome?

**DOI:** 10.3390/nu12082472

**Published:** 2020-08-17

**Authors:** Francisco J. Pérez-Cano

**Affiliations:** 1 Department of Biochemistry and Physiology, Faculty of Pharmacy and Food Science, University of Barcelona, 08028 Barcelona, Spain; franciscoperez@ub.edu; Tel.: +349-340-24505; 2 Nutrition and Food Safety Research Institute (INSA-UB), 08921 Santa Coloma de Gramenet, Spain

**Keywords:** microbiota, in utero development, diet, gestation, human milk oligosaccharides, health programming

## Abstract

This editorial aims to provide a concise summary of the factors involved in the dynamics of microbiome establishment and maturation. At the same time, it briefly updates the current knowledge and opens new questions in this regard. Many factors act as drivers of the microbiota’s development at both pre- and post-natal levels (e.g., maternal factors, antibiotic usage, type of delivery, dietary pattern, post-natal feeding type, etc.). However, it is interesting to research into its real impact, the relationship between these external modulators, and how to modulate them. The are great opportunities for new research in the field.

The mutualism between hosts and their microbiota is well-documented, especially in the gut. Human gut microbiota influences many physiological functions such as nutrition, metabolism and immunity, among others, both by direct and indirect mechanisms. Although the microbiota–host interaction is produced during the whole life period, it is especially important in early life, when alterations in composition or aberrant interactions can have consequences at later stages, by increasing the risk of several metabolic or immunologically driven disorders. For that reason, the factors involved in the microbial establishment and development of neonatal gut microbiota have received considerable interest in the last few years. 

In this regard, a remarkable review article published in “Nutrients” in 2018 elegantly described the factors affecting the development of the gastrointestinal (GI) microbiome during prenatal, perinatal, and postnatal life, along with their reciprocal relationship with GI tract maturation in neonates [1]. Particularly, Chong and collaborators made an overview of the GI development starting in utero and continuing after birth and the contribution of microbiota to this process. They also accurately addressed the environmental and extrinsic factors affecting gut microbiota development. In this context, the article showed a large number of well-documented evidences of the potential mechanisms involved in these factors’ actions. This revealing review concluded that it is essential to understand how each of these factors interact with each other to maintain intestinal homeostasis in infants, children, and adults. This knowledge may allow a better management of the modifiable conditions in order to enhance health, well-being and performance.

On the one hand, development of the GI tract including structure and function in early life is critical due to the exposure to a huge diversity of new foreign antigens. The gut initiates its formation in utero just few days after conception, although the key components of the mature gut are not present until the end of the second trimester of gestation, and it only becomes fully developed several months or years after birth. This GI tract development, both prenatally and postnatally, is suggested to work in parallel with the development of both the composition and activity of the microbiota [2], as well as with the mucosal immune system [3].

In their review, Chong et al. presented comprehensive evidences of the revolutionary change of paradigm from the “sterile gut before birth dogma” to the “in utero colonization hypothesis” [1]. This last hypothesis is based on studies demonstrating the presence of either the bacteria or its DNA as well as other bacterial cell products/components in the compartments involved in the possible bacterial maternal-fetal transfer (i.e., meconium, amniotic fluid or placenta) [2]. In addition, the importance of the amniotic fluid along with its fetal swallowing in the GI tract development is highlighted in terms of the intrauterine transmission of bacteria from the maternal microbiotas to the fetus. However, the background noise and false positives found when analyzing these extremely low bacterial abundances have to be taken into account. In this regard, although new approaches are being developed to quantify these low biomass samples [4], interpretation of these types of results should be cautious. Furthermore, a recent article by Rackaityte and collaborators showed that although viable bacteria are highly limited in the fetal intestine at mid-gestation, they can exert immunomodulatory functions [5]. In this context, and in line with what Collado and Segata stated in their editorial, the biological impact of these bacteria and their consequent short- and long-term health outcomes deserve further study [6]. 

With regard to the dynamics of the colonization of the infant intestine, it is crucial to highlight that the establishment of the microbiota after birth is highly variable in terms of composition due to enormous interindividual differences [2]. In fact, these differences—which in turn make it difficult to agree on a precise definition of an infant’s healthy intestinal microbiota—justify the necessity to understand the after-birth factors affecting such process. The above-mentioned review summarized the available results of the influence of the birth mode (cesarean vs. vaginal), impact of the type of infant feeding (breast milk or formula milk), antibiotic usage (mother, baby or both), and other environmental factors such as the extra uterine environment exposure or the intestinal oxygen levels, which seem to be different between term and preterm infants [1]. Besides this, the authors conclude that other factors apart from those included in the review could also be involved, including geography, host genetic factors, the influence of feeding the infant simultaneously with breast milk and formula milk, and even the composition of the infant formula.

Independently of “which” factors are involved in the infant’s microbiota development; the review outlines new questions. On the one hand, the first issue to address is “who”, focusing on the subject receiving the external influencing factor (i.e., the mother or the infant). Of course, many situations can influence the incipient microbiota development in the baby, but many others can have an impact on the mother too, affecting her microbiota composition and therefore the transmission to the new individual, both before and after birth. This last comment calls into question “when“ these environmental factors can have the greatest impact on the infant’s microbiota: prior to conception, during gestation or after birth. In line with this, the knowledge reflecting the maternal effect on the programming of the infant’s microbiota during gestation is increasing every day [7]. If we assume that maternal microbiota can be altered to promote a healthier impact on the infant, we have to think about “how”. Many strategies can be suggested, but there is no doubt that diet can have a role in shaping the microbiota in the mother and then transmit the changes to the infant [8]. It is also evident that maternal microbiota alterations can also have a negative impact on the descendance, and thus the maternal conditions in terms of health and disease should also be considered.

One of the key elements driving the postnatal infant’s microbiota is the breast milk, the bacterial composition of which is critical for this shaping action. At the same time, the bacterial dynamics of the triad mother’s milk–skin–baby’s oral cavity and the influencing microenvironmental factors are also under study. However, these, as well as other programming actions of breast milk, are not only due to its bacterial composition but also to its oligosaccharides content [9], along with the concomitant immune factors (e.g., secretory Immunoglobulin A) that help the establishment of the microbiota [10]. In this context, infant’s formulas nowadays do not only include certain probiotic bacteria, but also oligosaccharides mimicking breast milk composition [11]—the gold standard for infant’s development. 

In addition, new actors are also arising every day, for instance, the virome [12] and fungome [13] in both the neonatal intestine and breast milk, and their impacts on the infant are now attracting considerable interest.

In conclusion, the published review gives an in-depth idea of the state of the art in relation to the factors influencing the microbiota’s development. However, this editorial points out new recent evidence about the microbiota development in early life and the concern that many additional aspects deserve to be further studied and connected, searching for direct and indirect interactions. This knowledge may lead to the opportunity to make better health policies and recommendations for the baby´s health (derived from microbiota’s outcomes), both at an early age and in further stages of life (in the context of programming). 
